# Microglial Intracellular Ca^2+^ Signaling in Synaptic Development and its Alterations in Neurodevelopmental Disorders

**DOI:** 10.3389/fncel.2017.00069

**Published:** 2017-03-17

**Authors:** Yoshito Mizoguchi, Akira Monji

**Affiliations:** Department of Psychiatry, Faculty of Medicine, Saga UniversitySaga, Japan

**Keywords:** microglia, calcium signaling, BDNF, TRPC3 channels, proBDNF, oxytocin, synapse development, autism spectrum disorders

## Abstract

Autism spectrum disorders (ASDs) are neurodevelopmental disorders characterized by deficits in social interaction, difficulties with language and repetitive/restricted behaviors. Microglia are resident innate immune cells which release many factors including proinflammatory cytokines, nitric oxide (NO) and brain-derived neurotrophic factor (BDNF) when they are activated in response to immunological stimuli. Recent *in vivo* imaging has shown that microglia sculpt and refine the synaptic circuitry by removing excess and unwanted synapses and be involved in the development of neural circuits or synaptic plasticity thereby maintaining the brain homeostasis. BDNF, one of the neurotrophins, has various important roles in cell survival, neurite outgrowth, neuronal differentiation, synaptic plasticity and the maintenance of neural circuits in the CNS. Intracellular Ca^2+^ signaling is important for microglial functions including ramification, de-ramification, migration, phagocytosis and release of cytokines, NO and BDNF. BDNF induces a sustained intracellular Ca^2+^ elevation through the upregulation of the surface expression of canonical transient receptor potential 3 (TRPC3) channels in rodent microglia. BDNF might have an anti-inflammatory effect through the inhibition of microglial activation and TRPC3 could play important roles in not only inflammatory processes but also formation of synapse through the modulation of microglial phagocytic activity in the brain. This review article summarizes recent findings on emerging dual, inflammatory and non-inflammatory, roles of microglia in the brain and reinforces the importance of intracellular Ca^2+^ signaling for microglial functions in both normal neurodevelopment and their potential contributing to neurodevelopmental disorders such as ASDs.

## Introduction

Autism spectrum disorders (ASDs) are neurodevelopmental disorders characterized by deficits in social interaction, difficulties with language, and repetitive/restricted behaviors (Lai et al., 2014). The etiology of ASDs is still largely unclear, but both immune dysfunction and abnormalities in synaptogenesis have repeatedly been implicated as contributing to the disease phenotype (Edmonson et al., 2016).

Microglia are immune cells which are derived from progenitors that have migrated from the periphery and are from mesodermal/mesenchymal origin (Kettenmann et al., 2011). After invading the brain parenchyma, microglia transform into the “resting” ramified phenotype and are distributed in the whole brain. However, microglia revert to an ameboid appearance when they are activated in the disturbances including infection, trauma, ischemia, neurodegenerative diseases or any loss of brain homeostasis (Aguzzi et al., 2013; Cunningham, 2013). Microglia are the most active cytokine producing cells in the brain and can release many factors including pro-inflammatory cytokines (such as TNFα, IL-6), nitric oxide (NO) and neurotrophic factors (such as brain-derived neurotrophic factor, BDNF) when they are activated in response to immunological stimuli (Kato et al., 2013; Monji et al., 2013, 2014; Mizoguchi et al., 2014a; Smith and Dragunow, 2014). However, recent *in vivo* imaging has shown that microglia constantly use highly motile processes to survey their assigned brain regions and phagocyte pathogens and cellular debris even in their resting state, and are ready to transform to “activated” state in responses to injury, ischemia or autoimmune challenges in the brain (Wake et al., 2013). Microglia are also shown to sculpt and refine the synaptic circuitry by removing excess and unwanted synapses and be involved in the development of neural circuits or synaptic plasticity thereby maintaining the brain homeostasis (Schwartz et al., 2013; Hong et al., 2016). By extension, neurodevelopmental disorders such as ASDs might not need to involve a pathological gain in microglial function but simply a disruption of their physiological functioning in the regulation of synaptic circuits (Salter and Beggs, 2014; Ziats et al., 2015; Macht, 2016). This review article summarizes recent findings on emerging dual, inflammatory and non-inflammatory, roles of microglia in the brain and reinforces the importance of intracellular Ca^2+^ signaling for microglial functions in both normal neurodevelopment and their potential contributing to neurodevelopmental disorders such as ASDs.

## Inflammatory and Non-Inflammatory Roles of Microglia in ASDs

Numerous studies of serum cytokines demonstrated lower levels of transforming growth factor-β (TGF-β) and higher levels of macrophage inhibitory factor, leptin, interleukin 1β (IL-1β), IL-6, interferon-γ (IFN-γ) and IL-12 in various age groups of patients with ASDs (Goines and Ashwood, 2013). Cerebrospinal fluid (CSF) samples from patients with autism also showed an increase in pro-inflammatory macrophage chemoattractant protein 1 (MCP-1; Vargas et al., 2005) and TNF-α (Chez et al., 2007). Microglial abnormality could result from CNS or peripheral immune signals, such as auto-antibodies (Wills et al., 2009), or by peripheral chemokines/cytokines (such as IL-1β, IL-6 and TNF-α) up-regulated in autism (Gupta et al., 1998; Vargas et al., 2005).

The pioneer work by Vargas et al. (2005) and subsequent studies revealed an active neuroinflammatory phenotype of microglia in the post-mortem brains of patients with autism (Morgan et al., 2010). Marked changes in microglial morphology, accompanied by a unique profile of pro-inflammatory cytokines were seen in the cerebral cortex, white matter and cerebellum of patients with autism. Excessive microglia activation in young adults (age 18–31 years) affected by ASDs was also confirmed with PET using [11C]-(R)-PK11195. In this study, ASD brain regions showing increased binding potentials of the radiotracer included the cerebellum, midbrain, pons, fusiform gyri, and the anterior cingulate and orbitofrontal cortices. The most prominent increase was observed in the cerebellum (Suzuki et al., 2013). In the cerebellum, activated microglia were observed to be intimately associated with Purkinje cells undergoing apoptosis in cerebellar organotypic cultures during normal development. This could be consistent with a role for microglia in developmentally regulated neuronal death by promoting Purkinje cell apoptosis (Marín-Teva et al., 2004), an important physiological activity that could be impaired in autism.

A deficit in microglia/complement-mediated synaptic pruning might be fundamental to the cognitive effects associated with ASDs (Voineagu et al., 2011). The chemotactic/phagocytic activity of microglia could also be impaired, further aggravating the symptoms by insufficient clearance of debris (Derecki et al., 2013). The complement cascade, normally associated with removal of pathogens and cellular debris, is also crucial to microglial-mediated synaptic pruning and refinement of neuronal connectivity in the normal brain (Stephan et al., 2012). Evidence points to convergence on C3 and its microglial receptor C3 receptors (C3R). The initiator of the complement cascade is C1q, which induces C3 secretion via C4. The presence of C3 on unwanted synapses “tags” them for recognition by microglia to be eliminated. In addition, decreased C4 leading to reduced synaptic pruning in early life, mediated through reduced C3 synaptic tagging, is implicated in ASD-like behaviors (Estes and McAllister, 2015). Furthermore, mice deficient in the CX3CR1, a chemokine receptor expressed in the brain exclusively by microglia, have increased densities of immature synapses caused by delayed synaptic pruning, resulting in excessive and electrophysiologically immature synapses and deficits in functional connectivity (Zhan et al., 2014). Altogether, recent findings on emerging dual, inflammatory and non-inflammatory, roles of microglia in the brain suggest that abnormal secretion of inflammatory cytokines and abnormal or exaggerated execution of normal developmental microglial functions, including incorrect synaptic pruning, failure of phagocytosis of apoptotic neurons might be underlying mechanisms of neurodevelopmental disorders such as ASDs (Edmonson et al., 2016).

## Neuronal Intracellular Ca^2+^ Signaling Mediated by VGCCs and ASDs

The electrical activity of neurons (i.e., excitable cells) depends on a number of different types of voltage- or ligand-gated ion channels that are permeable to inorganic ions such as sodium, potassium, chloride and calcium. While the former three ions predominantly support the electrogenic roles, Ca^2+^ are different in that they can not only alter the membrane potential but also serve as important intracellular signaling entities by themselves. In the CNS, intracellular Ca^2+^ signaling regulates many different neuronal functions, such as cell proliferation, gene transcription and exocytosis at synapses (Berridge, 1998). In neurons, because the prolonged elevation of intracellular Ca^2+^ concentration ([Ca^2+^]i) is cytotoxic, [Ca^2+^]i is tightly regulated by intrinsic gating processes mediated by voltage-gated calcium channels (VGCCs) and NMDA receptors (NMDARs; Simms and Zamponi, 2014). In addition, dysregulation of neuronal Ca^2+^ signaling have been linked to neurodevelopmental disorders including ASDs (Krey and Dolmetsch, 2007). Ca_V_1.3 channels are a major class of L-type VGCCs which constitute an important calcium entry pathway implicated in the regulation of spine morphology and then contribute to the rhythmicity of brain (Stanika et al., 2016). In the brain, VGCCs are vital for coupling of neuronal excitation-transcription, synaptic plasticity and neuronal firing, and *de novo* missense mutation A760G of Ca_V_1.3 channels has been linked to ASDs (Pinggera et al., 2015). Ca_V_1.3 channels employ two major forms of feedback regulation, voltage-dependent inactivation (VDI) and Ca^2+^-dependent inactivation (CDI). Limpitikul et al. (2016) recently found that introduction of missense mutation A760G to Ca_V_1.3 severely suppressed the CDI but also potentiated the VDI of Ca_V_1.3 channels, suggesting that deficits of these two feedback regulation appear to increase the [Ca^2+^]i, thus potentially disrupting both neuronal development and synapse formation, ultimately leading to ASDs. There are many other reports showing that functional mutations in genes encoding VGCCs can lead to ASDs (Splawski et al., 2004; Li et al., 2015). In addition, disruption of the BK_Ca_ gene *KCNMA1* which encodes the α-subunit of the large conductance Ca^2+^-activated K^+^ channel (BK_Ca_) led to both haplo-insufficiency and reduced BK_Ca_ activity (Laumonnier et al., 2006). Thus, the reported decrease in BK_Ca_ channel activity, together with the reduced inactivation of VGCCs in autistic patients, suggests that ASDs are caused by abnormally sustained increases in intracellular Ca^2+^ levels (Krey and Dolmetsch, 2007).

## Microglial Intracellular Ca^2+^ Signaling and Importance of TRP Channels

Elevation of [Ca^2+^]i is also important for the activation of microglia, including proliferation, migration, ramification, de-ramification and release of NO, proinflammatory cytokines and BDNF (Kettenmann et al., 2011). In addition, disruption of microglial Ca^2+^ homeostasis triggers activation of death programs, which are regulated by the microglia activation status. Treatment of primary cultured microglial cells with thapsigargin or ionomycin induced apoptosis, whereas the same agents applied to lipopolysaccharide (LPS)-activated microglia resulted in necrotic cell death (Nagano et al., 2006). Both apoptotic and necrotic pathways are regulated by [Ca^2+^]i because the treatment of cultures with BAPTA-AM reduced microglial cell death (Nagano et al., 2006). However, in microglial cells, an application of high [K^+^]out or glutamate does not elevate [Ca^2+^]i. This observation is supported by the fact that both VGCCs and NMDARs are not expressed in microglia (Kettenmann et al., 2011). For electrically non-excitable cells including microglia, the primary source of intracellular Ca^2+^ is the release from intracellular Ca^2+^ stores and the entry through the ligand-gated and/or store operated Ca^2+^ channels (Möller, 2002). Microglia contain at least two types of intracellular Ca^2+^ stores: the endoplasmic reticulum (ER) and mitochondria. The main route for the generation of intracellular Ca^2+^ signaling is associated with inositol 1,4,5-trisphosphate (InsP3) receptors on the ER membrane. Stimulation of G protein-coupled metabotropic or tyrosine kinase receptors results in the activation of the phospholipase C (PLC), production of two second messengers including the diacylglycerol (DAG) and the InsP3 and the release of Ca^2+^ from the ER. Importantly, the depletion of ER activates the store-operated Ca^2+^ entry (SOCE), known as a capacitative Ca^2+^ influx, mediated by plasmalemmal channels such as calcium release-activated Ca^2+^ (CRAC) channels and/or transient receptor potential (TRP) channels (Parekh and Putney, 2005). In addition, STIM1, one of ER membrane proteins, senses the filling state of ER Ca^2+^ and delivers the ER to the plasma membrane where it directly activates Orai1/CRAC channels, thereby facilitating the re-uptake of Ca^2+^ to ER through the sarco(endo)plasmic reticulum Ca^2+^-ATPases (SERCA). The concentration of Ca^2+^ in the ER is precisely controlled by SERCA. Recently, Schmunk et al. (2015) found that dysregulation of InsP3/ER signaling in primary, untransformed skin fibroblasts derived from patients with Fragile X (FXS) or tuberous sclerosis syndromes. This suggests that ASDs might also affect the status of the ER-Ca^2+^ store in microglial cells.

The influx of Ca^2+^ through the TRP channels could play some important roles in many inflammatory processes including the activation of microglia (Nilius and Szallasi, 2014). There are seven transient receptor potential canonical (TRPC) channels in mammalian species. Among them, TRPC2 is a pseudogene in humans. The remaining members of the TRPC subfamily are classified into three groups according to sequence homology, TRPC1, canonical TRPC3/C6/C7 and TRPC4/C5. Quantitative comparisons of mRNA expression using real-time RT-PCR showed that TRPM7 > TRPC6 > TRPM2 > TRPC1 > TRPC3 ≥ TRPC4 > TRPC7 > TRPC5 > TRPC2, where “>” denotes a significant difference from the preceding gene, and “≥” indicates a non-significant difference, in microglial cells cultured from rats (Ohana et al., 2009).

## Importance of BDNF Signaling in ASDs

BDNF, one of the neurotrophins, has various important roles in cell survival, neurite outgrowth, neuronal differentiation and gene expression in the brain (Thoenen, 1995; Park and Poo, 2013). BDNF is most abundantly expressed in the hippocampus and cerebral cortex and is also involved in the pathophysiology of psychiatric disorders (Sen et al., 2008). Two meta-analysis studies recently showed that neonates diagnosed with ASDs later in life had no association with blood levels of BDNF, while children in the ASD group demonstrated significantly increased BDNF levels compared with healthy controls (Qin et al., 2016; Zheng et al., 2016). These suggest that peripheral BDNF levels might serve as a potential biomarker for the diagnosis of ASDs and further studies are needed to clarify the causal relationship between the symptoms of ASDs and peripheral levels of BDNF.

BDNF binds to the tropomyosin-related kinase B (TrkB) receptor and induces the activation of intracellular signaling pathways, including PLC-γ, phosphatidylinositol 3-kinase (PI3K) and mitogen activated protein kinase-1/2 (MAPK-1/2; Patapoutian and Reichardt, 2001). BDNF rapidly activates the PLC pathway, leading to the generation of InsP3 and the mobilization of intracellular Ca^2+^ from the ER (Mizoguchi et al., 2003a,b). TRPC3 channels are shown to be necessary for BDNF to increase the density of dendritic spines in rodent hippocampal CA1 pyramidal neurons (Amaral and Pozzo-Miller, 2007). Rett syndrome (RTT) is caused by loss-of-function mutations in *MECP2*, encoding methyl-CpG-binding protein 2 (Amir et al., 1999). The TRPC3 mRNA and protein levels are lower in CA3 pyramidal neurons of symptomatic *Mecp2* mutant mice and chromatin immunoprecipitation (ChIP) identified *Trpc3* as a target of MeCP2 transcriptional regulation. BDNF mRNA and protein levels are also lower in *Mecp2* mutant hippocampus and dentate gyrus granule cells, which is reflected in impaired activity-dependent release of endogenous BDNF. These results identify the gene encoding TRPC3 channels as a MeCP2 target and suggest a potential therapeutic strategy to boost impaired BDNF signaling in RTT (Li et al., 2012).

## Possible Involvement of Microglial Intracellular Ca^2+^ Signaling Modulated by BDNF in ASDs

In the rodent brain, microglial cells express BDNF mRNA (Elkabes et al., 1996) and secrete BDNF following stimulation with LPS (Nakajima et al., 2001). BDNF released from activated microglia then induces the sprouting of nigrostriatal dopaminergic neurons (Batchelor et al., 1999), causing a shift in the neuronal anion gradient (Coull et al., 2005), or promotes the proliferation and survival of microglia themselves (Zhang et al., 2003). In addition, Parkhurst et al. (2013) showed that the Cre-dependent removal of BDNF from microglia induces deficits in multiple learning tasks mediated by reduction in learning-dependent spine elimination/formation. These suggest that microglia serve important physiological functions in learning and memory by promoting learning-related synapse formation through the BDNF signaling.

We have reported that BDNF induced a sustained increase in [Ca^2+^]i through binding with the truncated tropomyosin-related kinase B receptor (TrkB-T1), resulting in activation of the PLC pathway and SOCE in rodent microglial cells. Sustained activation of SOCE occurred after a brief BDNF application and contributed to the maintenance of sustained [Ca^2+^]i elevation. Pretreatment with BDNF significantly suppressed the release of NO from activated microglia. Additionally, pretreatment of BDNF suppressed the IFN-γ-induced increase in [Ca^2+^]i, along with a rise in basal levels of [Ca^2+^]i in rodent microglial cells (Mizoguchi et al., 2009). Thereafter, we observed that TRPC3 channels contribute to the maintenance of BDNF-induced sustained intracellular Ca^2+^ elevation. Immunocytochemical technique and flow cytometry also revealed that BDNF rapidly up-regulated the surface expression of TRPC3 channels in rodent microglial cells. BDNF-induced up-regulation of surface expression of TRPC3 channels also depends on activation of the PLC pathway, as previously shown by others (van Rossum et al., 2005). In addition, pretreatment with BDNF suppressed the production of NO induced by TNFα, which was prevented by co-administration of a selective TRPC3 inhibitor, Pyr3. These suggest that TRPC3 channels could be important for the BDNF-induced suppression of the NO production in activated microglia. We first showed direct evidence that rodent microglial cells are able to respond to BDNF and TRPC3 channels could also play important roles in microglial functions. Hall et al. (2009) have previously demonstrated the implication of the basal level of [Ca^2+^]i in the activation of rodent microglia, including NO production. BDNF-induced elevation of basal levels of [Ca^2+^]i could regulate the microglial intracellular signal transduction to suppress the release of NO induced by IFN-γ (Hoffmann et al., 2003; Mizoguchi et al., 2009). We observed that pretreatment with BDNF also suppressed the production of NO in murine microglial cells activated by TNFα, which was prevented by co-administration of Pyr3. We also found that pretreatment with both BDNF and Pyr3 did not elevate the basal [Ca^2+^]i in rodent microglial cells. These suggest that BDNF-induced elevation of basal levels of [Ca^2+^]i mediated by TRPC3 channels could be important for the BDNF-induced suppression of NO production in rodent microglial cells. Although the mechanism underlying the activation of TRPCs via PLC stimulation is still not completely resolved, TRPC3, like TRPC6 and TRPC7, can be activated directly by DAG. The trafficking of TRPC3 channels to the plasma membrane depends on interactions with Cav-1, Homer1, PLC-γ, VAMP2 and RFN24 (de Souza and Ambudkar, 2014). In addition, phagocytic activity is suppressed by pharmacological inhibitors of SOCE in murine microglial cells (Heo et al., 2015). Altogether, these suggest that BDNF might have an anti-inflammatory effect through the inhibition of microglial activation and TRPC3 could play important roles in not only inflammatory processes but also formation of synapse through the modulation of microglial phagocytic activity in the brain. We need additional studies to identify the molecular mechanisms that determine the trafficking and activity of TRPC3 channels and what underlies the BDNF-induced up-regulation of surface TRPC3 channels in these mechanisms (Mizoguchi et al., 2014a,b).

Using multiple models including individual’s dental pulp cells (DPCs), neural cells derived from induced pluripotent stem cell (iPSCs) and mouse models, Griesi-Oliveira et al. (2015) recently reported that loss-of-function mutations of TRPC6 is a novel predisposing factor for ASDs, suggesting that dysfunction of Ca^2+^ signaling mediated by TRPC6 contributes to altered neuronal development, neuronal morphology and synaptic function in ASDs. It is not well known whether TRPC6 channels of microglia also serve important physiological roles in the alteration of synaptic function in ASDs.

## Future Prospects

**Figure 1:**
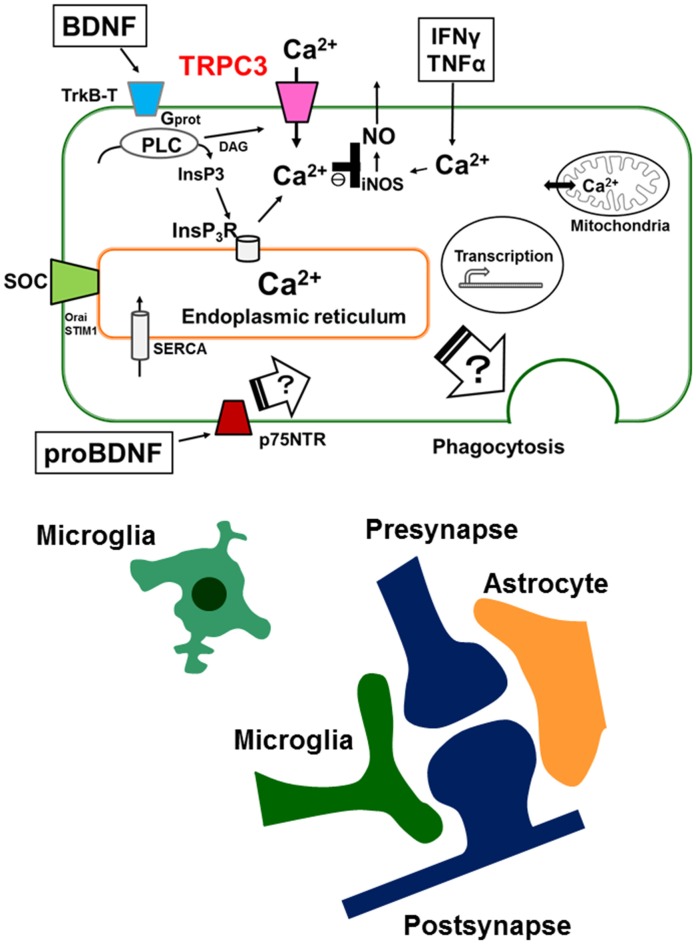
**Schematic illustration representing the microglial intracellular Ca^2+^ signaling mediated by canonical transient receptor potential 3 (TRPC3) channels and the tripartite synapse.** In microglia, brain-derived neurotrophic factor (BDNF) induces a sustained increase in [Ca^2+^]i through binding of the truncated TrkB receptors (TrkB-T), resulting in activation of the phospholipase C (PLC) pathway. Up-regulation of cell surface TRPC3 channels occurs after a brief treatment with BDNF and contributes to the maintenance of BDNF-induced sustained intracellular Ca^2+^ elevation. BDNF-induced elevation of basal levels of [Ca^2+^]i mediated by TRPC3 channels could be important for the BDNF-induced suppression of nitric oxide (NO) production induced by TNFα or IFNγ. Microglial intracellular Ca^2+^ signaling is also important for microglial functions such as phagocytosis in the brain. The tripartite synapse consists of the presynaptic (glutamatergic) terminal, postsynaptic terminal, astrocytes and microglia. Dysregulation of normal microglial functions including incorrect synaptic pruning, failure of phagocytosis of apoptotic neurons and abnormal secretion of inflammatory cytokines might be underlying mechanisms of neurodevelopmental disorders such as autism spectrum disorders (ASDs). On the other hand, the effects of proBDNF on microglial functions are not fully understood. Further work will be needed to elucidate the role of proBDNF on microglial cells by focusing on intracellular Ca^2+^ signaling mediated by TRPC channels.

Elevation of intracellular Ca^2+^ is important for the activation of microglial cell functions, including proliferation, release of NO, cytokines and BDNF. It has been shown that alteration of intracellular Ca^2+^ signaling underlies the pathophysiology of neurodevelopmental disorders including ASDs. BDNF induces a sustained intracellular Ca^2+^ elevation through the upregulation of the surface expression of TRPC3 channels in rodent microglial cells. Microglial cells are able to respond to BDNF, which may be important for the regulation of inflammatory responses, and may also be involved in the normal development of CNS. BDNF is first synthesized as proBDNF protein. ProBDNF is then either proteolytically cleaved intracellularly or by extracellular proteases, such as metalloproteinases and plasmin, to mature BDNF. Interestingly, interaction of mature neurotrophins with Trk receptors leads to cell survival, whereas binding of proBDNF to p75NTR leads to apoptosis. In addition, mature BDNF and proBDNF facilitates long-term potentiation (LTP) and long-term depression (LTD) at the hippocampal CA1 synapses, respectively. Thus, Trk and p75NTR preferentially bind mature- and pro-neurotrophins, respectively, to elicit opposing biological responses in the CNS (Greenberg et al., 2009). Indeed, a recently published report shows that pruning of spines promoted by proBDNF is mediated by the p75NTR-RhoA, while maturation of spines induced by BDNF is through the stimulation of TrkB-Rac1 signaling (Orefice et al., 2016). However, the effects of proBDNF on microglial cells are not fully understood. Thus, further work will be needed to elucidate the role of proBDNF on microglial cells by focusing on TRPC channels (Figure 1).

Oxytocin (OT) is a pituitary neuropeptide hormone synthesized from the paraventricular and supra-optic nuclei within the hypothalamus. Like other neuropeptides, OT can modulate a wide range of neurotransmitter and neuromodulator activities. OT is secreted into the systemic circulation to act as a hormone, thereby influencing several body functions. OT plays a pivotal role in parturition, milk let-down and maternal behavior and has been demonstrated to be important in the formation of pair bonding between mother and infants as well as in mating pairs. Furthermore, OT has been proven to play a key role in the regulation of several behaviors associated with neuropsychiatric disorders, including social interactions, social memory response to social stimuli, decision-making in the context of social interactions, feeding behavior, emotional reactivity, etc. An increasing body of evidence suggests that dysregulation of the oxytocinergic system might be involved in the pathophysiology of neurodevelopmental disorders such as ASDs (Romano et al., 2016). Using functional magnetic resonance imaging, single-dose intranasal administration of OT was shown to improve the frequency of the nonverbal information-based judgments with a shorter response time and the brain activity of the medial prefrontal cortex in participants with ASDs (Watanabe et al., 2014). Thus, there is a significant potential for OT to ameliorate some aspects of the persistent and debilitating social impairments in individuals with ASDs (Alvares et al., 2016). Although there is no clinical use of minocycline in ASDs, prenatal minocycline treatment can alter the expression of PSD-95 and ameliorate abnormal mother-infant communication in oxytocin receptor (Oxtr)-deficient mice (Miyazaki et al., 2016). This finding suggests that minocycline has a therapeutic potential for the development of OT/Oxtr-mediated ASD-like phenotypes (Nakagawa and Chiba, 2016). In addition, OT suppressed both the mRNA expression of TNFα, IL-1β, COX-2 and iNOS and the elevation of [Ca^2+^]i in LPS-stimulated microglia cells (Yuan et al., 2016). These suggest that OT would be a potential therapeutic agent for alleviating neuro-inflammatory processes in ASDs. However, the effects of OT on microglial intracellular Ca^2+^ signaling are not fully understood. Thus, it will be important to study the effect of OT on microglial cells especially by focusing on TRPC channels.

## Conclusions

There is increasing evidence suggesting that pathophysiology of neurodevelopmental disorders is related to the inflammatory responses mediated by microglial cells. In addition, recent advances in the understanding of microglial functions suggest an important role for these cells in the normal development of CNS in addition to their traditional role as immune cells of the brain. Dysregulation of normal microglial functions such as regulation of programmed cell death and/or synaptic pruning is supposed to be increasingly implicated in ASDs associated with cognitive deficits. These findings have resulted in a new model of the synapse as “tripartite,” recognizing the important role of not just neurons and astrocytes but also microglia in the normal physiological function of the brain. We now need to explore the emerging dual, inflammatory and non-inflammatory, roles of microglia in the brain and recent findings reinforce the importance of intracellular Ca^2+^ signaling for microglial functions in both normal neurodevelopment and their potential contributing to neurodevelopmental disorders such as ASDs.

## Author Contributions

YM and AM wrote this article.

## Conflict of Interest Statement

The authors declare that the research was conducted in the absence of any commercial or financial relationships that could be construed as a potential conflict of interest.
